# The influence of immune factors on bone metabolism in autoimmune thyroid diseases

**DOI:** 10.3389/fendo.2026.1765560

**Published:** 2026-02-26

**Authors:** Wanting Zhao, Ling Cui, Hui Si, Xin Hou

**Affiliations:** Department of Geriatrics Endocrinology, The First Affiliated Hospital of China Medical University, Shenyang, Liaoning, China

**Keywords:** autoimmune thyroid diseases, B lymphocytes, bone metabolism, macrophages, T lymphocytes

## Abstract

Autoimmune thyroid diseases (AITD) primarily includes Graves’ disease (GD) and Hashimoto’s thyroiditis (HT), which share common features such as the presence of thyroid autoantibodies and lymphocyte infiltration. Studies have shown that immune cells and their secreted cytokines in AITD not only participate in thyroid autoimmune responses but also regulate osteoblast and osteoclast activity in bone metabolism, affecting bone resorption and formation. Additionally, thyroid autoantibodies may directly affect bone metabolism through mechanisms like oxidative stress. This review emphasizes the dual role of immune factors in AITD in bone metabolism, providing new perspectives for understanding the relationship between these two diseases.

## 1 Introduction

Autoimmune thyroid diseases (AITD) represents a group of autoimmune conditions that primarily affect the thyroid gland. Clinically, Graves’ disease (GD) and Hashimoto’s thyroiditis (HT) are the most prevalent forms of AITD. A hallmark immunopathological feature of AITD is lymphocytic infiltration of the thyroid gland, mainly composed of T and B lymphocytes, accompanied by the presence of circulating thyroid autoantibodies, including TSH receptor antibodies (TRAb), thyroid peroxidase antibodies (TPOAb), and thyroglobulin antibodies (TgAb).

Bone metabolism refers to a dynamic physiological process that maintains bone homeostasis through the coordinated activities of bone formation and bone resorption. In response to microdamage or mechanical stimulation of the bone surface, osteoblasts secrete receptor activator of nuclear factor-κB ligand (RANKL). RANKL binds to its receptor, receptor activator of nuclear factor-κB (RANK), on osteoclast precursors, thereby activating multiple downstream signaling pathways that promote osteoclast differentiation and maturation, ultimately initiating bone resorption. Following bone resorption, osteoclasts undergo apoptosis and release various growth factors that recruit and activate osteoblast precursors. Concurrently, osteoblasts secrete osteoprotegerin (OPG) to inhibit excessive bone resorption and synthesize new bone matrix to promote bone formation. The relative ratio of OPG to RANKL dynamically regulates osteoclast activity, thereby maintaining equilibrium between bone resorption and bone formation. When this balance is disrupted, it can cause decreased bone mass, and destruction of bone microstructure, leading to increased bone fragility and a metabolic bone disorder characterized by susceptibility to fractures, termed osteoporosis.

Research by Polovina et al. demonstrated that TPOAb, independent of thyroid stimulating hormone (TSH), is significantly correlated with an elevated risk of fractures (1). Research in the elderly population indicates that TPOAb positivity is an independent risk factor for abnormal bone mass, whereas TgAb shows no significant correlation with abnormal bone mass (2). Wang et al. shows TPOAb and TgAb levels are negatively correlated with bone mineral density (BMD), and TPOAb is an independent influencing factor for diabetic osteoporosis (3). Su et al. reported that serum TRAb levels in GD patients showed a significant positive correlation with three bone turnover markers, This indicates that higher TRAb titers are associated with more active bone metabolism (4). Research in postmenopausal GD patients revealed a significant inverse relationship between BMD and TRAb (5). However, Some studies have presented contrary conclusions. Mira Siderova et al. investigated the factors affecting bone health in women with Graves’ thyrotoxicosis and Graves’ orbitopathy (GO), finding a significant positive correlation between TRAb and BMD, while TRAb was negatively correlated with fracture risk (6). This indicates that thyroid autoimmunity could influence bone metabolism. The pathogenesis of thyroid autoimmunity and bone immunology is intricate, involving multiple immune cells, cytokines, and chemokines. Identical cytokines can influence various tissues and organs. We will provide a review of the relationship between AITD and osteoporosis from an immunological standpoint.

## 2 Immune mechanisms of autoimmune thyroid diseases

The hallmark of GD is the positivity of serum TRAb, along with clinical symptoms including excessive sweating, irritability, and weight loss. The thyroid gland in GD patients shows diffuse enlargement to varying extents, with follicular epithelial cell hyperplasia appearing as tall columnar or cuboidal shapes, reduced or absent colloid in the follicular lumen, and lymphocyte infiltration related to lymphoid germinal centers between follicles. The infiltrating lymphocytes are mainly T lymphocytes, with a minority of B lymphocytes and macrophages. Lymphocytes identify the TSH-R antigen in thyroid cells and generate corresponding antibodies, TRAb. Depending on their actions on TSH-R, TRAb antibodies are categorized into: thyroid stimulation antibody (TSAb);thyroid stimulation blocking antibody (TSBAb); and neutral antibodies. The features of HT include markedly increased serum titers of TPOAb and TgAb, along with diffuse thyroid enlargement. The normal follicular architecture of the thyroid is largely replaced by infiltrating lymphocytes, plasma cells, macrophages, and their lymphoid germinal centers. The infiltrating lymphocytes consist of T lymphocytes and B lymphocytes, which, together with cytokines, trigger intracellular apoptotic pathways, resulting in thyroid cell apoptosis and follicular destruction. Hypothyroidism develops when 90% of the thyroid follicles are damaged (7).

Infiltrating T cells within the thyroid gland differentiate into helper T (Th) cell subsets and Regulatory T (Treg) cells with distinct functional profiles (8). Th1 lymphocytes predominantly produce interferon-γ (IFN-γ), whereas Th17 lymphocytes secrete interleukin-17 (IL-17). Tregs are central in antagonizing autoimmunity. These cytokines activate fibroblasts and macrophages, thereby contributing to autoimmune-mediated tissue damage (9). Furthermore, activated Th cells provide stimulatory signals to B lymphocytes. Following antigen recognition via surface membrane immunoglobulins, B cells differentiate into plasma cells that produce autoantibodies, including TRAb, TgAb, TPOAb. Macrophages are one of the innate immune cells present in thyroid tissue. Damaged cells and immune cells release various pro-inflammatory cytokines, which promote macrophage activation (10).

## 3 The impact of immune factors on bone metabolism

In a physiological state, a dynamic equilibrium exists between bone formation driven by osteoblasts and bone resorption driven by osteoclasts, contributing to the maintenance of normal bone mass and BMD. Osteoblasts express RANKL, which binds to RANK on osteoclasts to regulate their differentiation, and also produce OPG. Upon binding of RANKL to RANK, multiple downstream signaling pathways are activated, promoting osteoclast differentiation and activation. This process is inhibited by OPG, which functions as a decoy receptor that blocks the RANK–RANK interaction. The RANK/RANKL/OPG axis plays a critical role in the molecular mechanisms of osteoporosis (11). When pro-inflammatory cytokines act on this axis and lead to excessive osteoclast formation, bone resorption increases, ultimately resulting in reduced bone mass, impaired bone tissue microstructure, and the occurrence of osteoporosis.

### 3.1 The role of T lymphocytes in bone metabolism

T lymphocytes can promote bone resorption through direct or indirect actions on the RANK/RANKL/OPG axis. Firstly, T lymphocytes can directly express RANKL, which binds to RANK on osteoclasts. Activation of RANK on osteoclasts sends signals into the cell via “adaptor proteins.” Among these adaptor proteins is TRAF6, enabling RANKL to activate various signaling pathways, including the transcription factor NF-κB and protein kinases like JNK and c-Src. These pathways facilitate osteoclast maturation and their ability to resorb bone (12).

Additionally, T lymphocytes can also indirectly induce bone resorption by secreting cytokines that affect the RANK/RANKL/OPG axis. IFN-γ secreted by Th1 cells can induce macrophages to produce cytokines such as IL-6, TNF-α, and IL-1, which promote bone resorption (13).Th17 cells secret IL-17 which promotes osteoclastogenesis by increasing the expression of osteoclastogenic cytokines like RANKL, TNF-α, IL-1, IL-6, and IL-8, while also suppressing late-stage osteoblast differentiation through downregulation of Wnt pathway genes, such as Axin2, Wisp1, and Bmp4 (14). Additionally, IL-17 activates NF-κB in osteoblasts, impairing their differentiation capacity (15).

**Figure 1:**
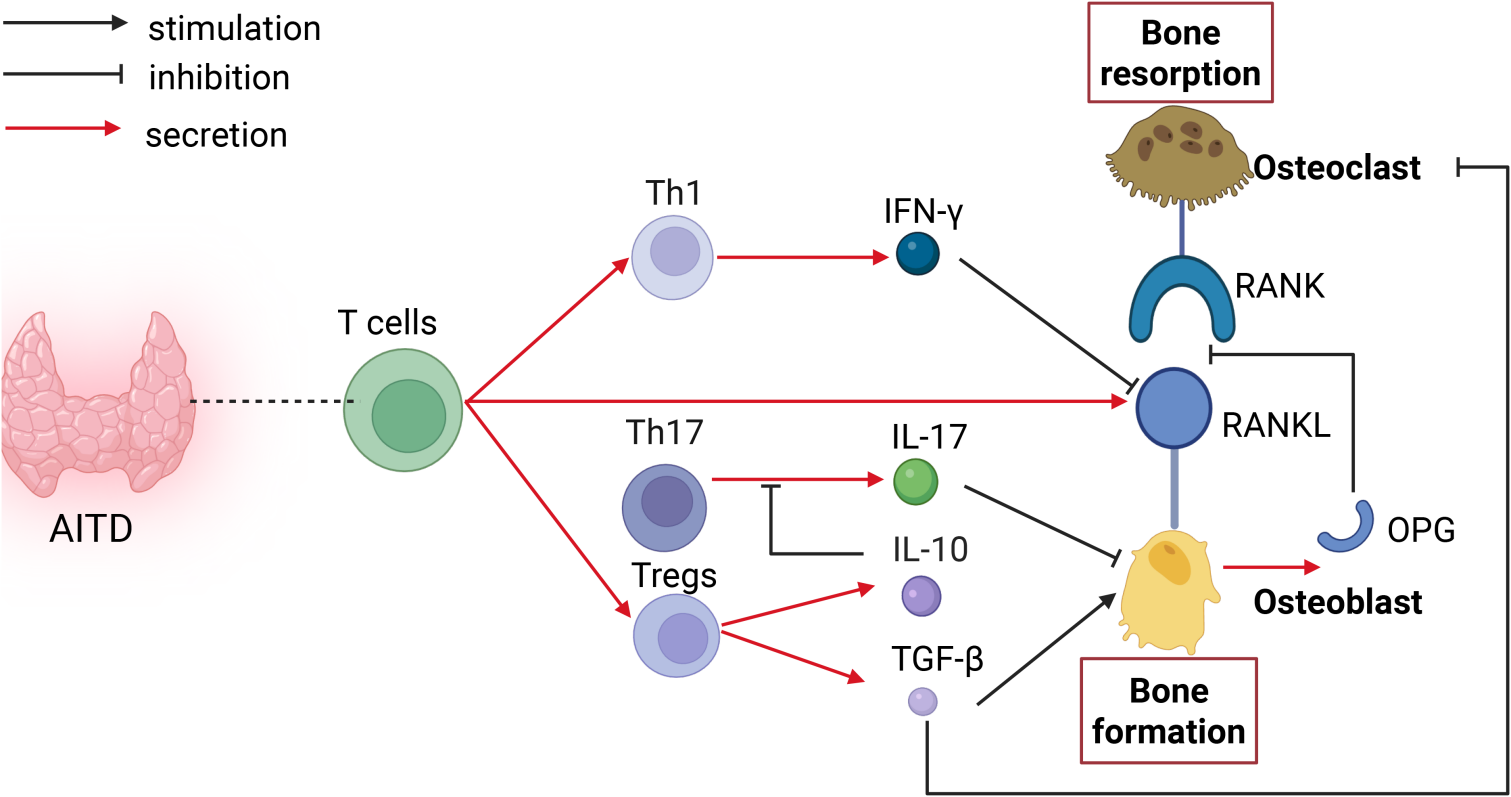
Impact of T cells on bone metabolism in AITD. T cells influence bone metabolism through direct secretion of RANKL (promoting resorption) and via cytokines produced by their subsets. Th1-derived IFN-γ inhibits RANKL expression to support bone formation, whereas Th17-derived IL-17 suppresses osteoblast differentiation, impairing bone formation. Treg cells exert dual regulatory effects: they inhibit the bone-resorptive Th17 subset via IL-10, and secrete TGF-β to directly promote osteoblast differentiation while inhibiting osteoclastogenesis, collectively favoring bone formation.

Moreover, T lymphocytes can play an anti-resorptive role:Th1 cells release interferon-γ, which accelerates the degradation of TRAF6, effectively suppressing the activation of RANKL-induced transcription factors NF-κB and JNK, thereby preventing activated T cells from causing significant bone destruction during inflammation and exerting anti-resorptive effects (16–18). Tregs are involved in bone metabolism through the secretion of IL-10 and TGF-β. IL-10 suppresses Th17 function, exerting a bone-forming effect (19).TGF-β exerts a dual role in bone metabolism: it promotes bone formation by stimulating osteoblast proliferation, differentiation, maturation, and activity, while simultaneously inhibiting bone resorption by suppressing osteoclast differentiation and formation (20) (as shown in **Figure 1**).

### 3.2 The role of B lymphocytes in bone metabolism

**Figure 2:**
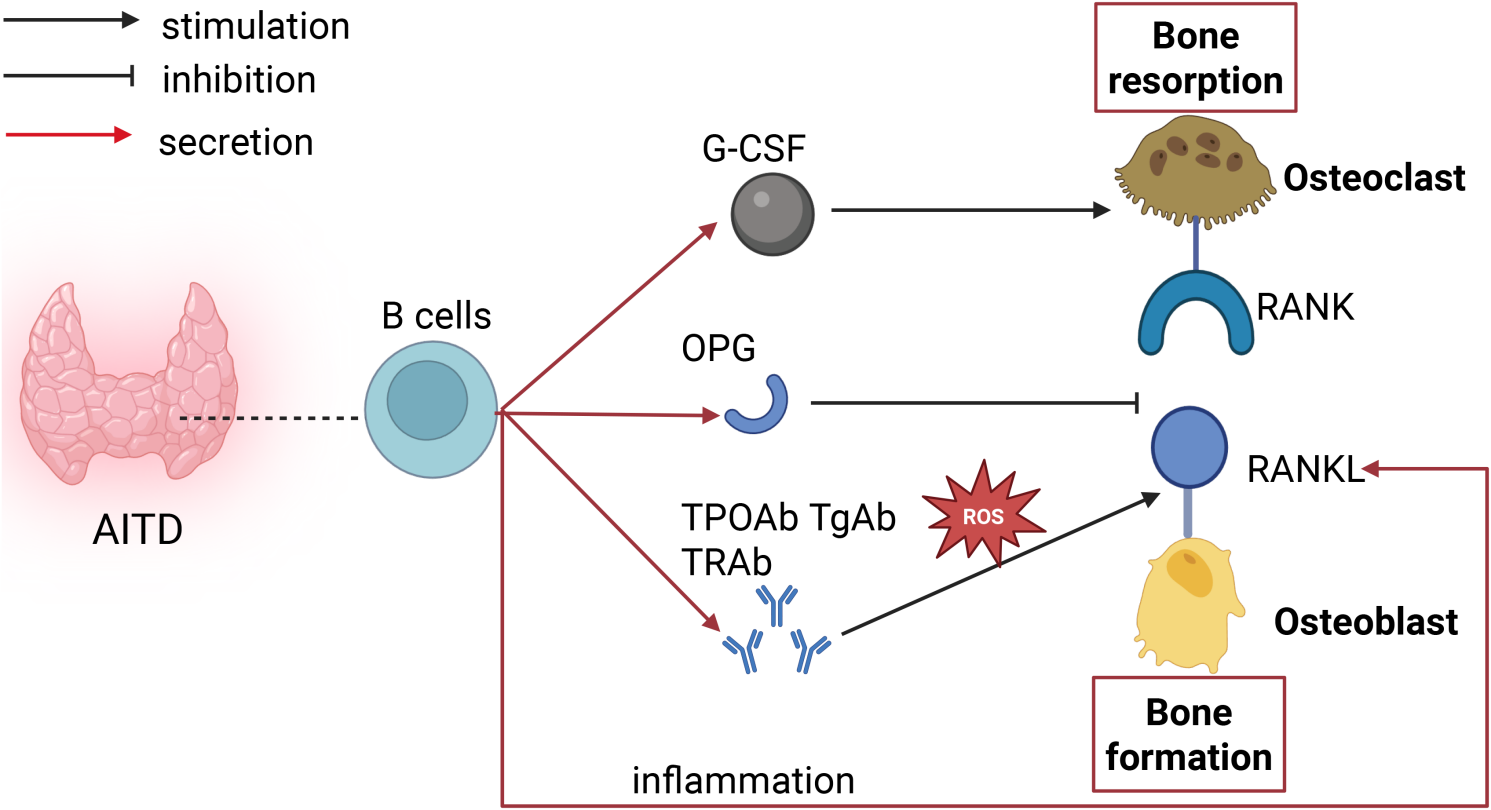
Impact of B cells on bone metabolism in AITD. B cells secrete G-CSF to stimulate osteoclast generation. Antibodies produced by B cells can upregulate RANK expression through oxidative stress, enhancing bone resorption. B cells secrete OPG, which inhibits the binding of RANK to RANKL and suppresses bone resorption. B cells secrete RANKL, which binds to RANK and promotes bone resorption.

B lymphocytes can secrete granulocyte colony-stimulating factor (G-CSF), which stimulates the proliferation of osteoclast precursors, thereby increasing osteoclast formation. Besides G-CSF, activated B lymphocytes excessively secrete RANKL under inflammatory conditions, binding to RANK and triggering osteoclast formation. Research has also proposed that some B lymphocytes might act as osteoclast precursors, possessing the ability to differentiate into osteoclasts, thus elevating osteoclast generation. Moreover, B lymphocytes also play a role in inhibiting bone resorption. B lymphocytes activate the ERK and NF-κB signaling pathways by secreting C-C motif chemokine ligand 3 (CCL3) and TNF, inhibiting osteoblast differentiation and thereby suppressing bone formation (21–23). B lymphocytes generate about 40-60% of the total bone marrow-derived OPG, suppressing osteoclast differentiation (as shown in **Figure 2**).

### 3.3 The role of macrophages in bone metabolism

**Figure 3:**
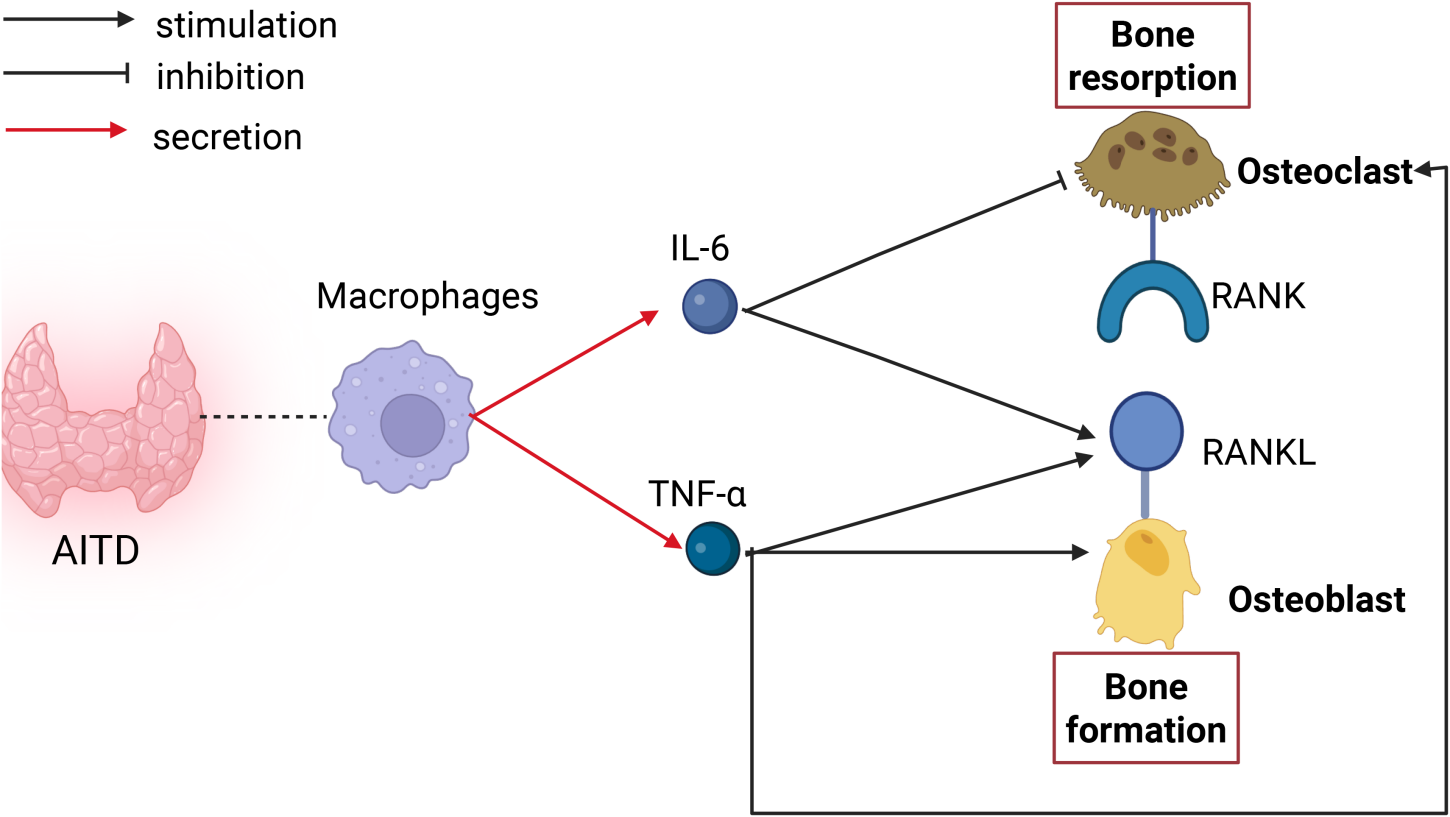
Impact of macrophages on bone metabolism in AITD. Macrophages can secrete IL-6 to stimulate osteoblasts to produce RANKL, thereby promoting increased bone resorption;IL-6 can also inhibit osteoclast differentiation, thus exerting an anti-resorptive effect. Macrophages can secrete TNF-α to act directly on osteoblasts and osteoclasts, increasing the expression of RANKL and RANK, and promoting bone resorption; TNF-α can also promote RANKL expression to exert bone-resorptive effects.

Macrophages can influence bone metabolism by secreting cytokines such as IL-6, TNF-α, and IL-1 (24). IL-6 enhances osteoclast formation via direct and indirect mechanisms (25). Firstly, IL-6 directly stimulates osteoclast formation via a RANKL-independent mechanism, but the precise mechanism remains unclear. IL-6 further indirectly promotes osteoclast formation by activating STAT3 via JAK, leading to RANKL production in stromal cells and osteoblasts. RANKL binds to RANK on osteoclasts, enhancing bone resorption. TNF-α directly increases RANK expression in osteoclasts and stimulates RANKL production in osteoblasts, contributing to osteoporosis development. TNF-α activates the NF-κB transcription factor, leading to c-Fos expression and osteoclast formation. Furthermore, TNF-α works synergistically with RANKL and M-CSF to stimulate osteoclastogenesis, greatly amplifying osteoclast activity (26, 27). Additionally, research indicates that TNF-α pretreatment sensitizes M-CSF-induced M2 macrophages to pro-inflammatory M1 polarization, expanding the osteoclast precursor pool and enhancing osteoclastogenic potential (28). TNF-α further promotes sclerostin (SOST) expression, stimulating RANKL expression in osteocytes and augmenting osteoclast formation (14). Moreover, T lymphocytes can play an anti-resorptive role: IL-6 inhibits osteoclast precursor differentiation into osteoclasts by inducing prostaglandin E2 production in chondrocytes, thereby acting as an anti-resorptive agent (29). In inflammatory processes, T cells release interferon-γ, which accelerates the degradation of TRAF6, effectively suppressing the activation of RANKL-induced transcription factors NF-κB and JNK, thereby preventing activated T cells from causing significant bone destruction during inflammation and exerting anti-resorptive effects (16–18) (as shown in **Figure 3**).

## 4 The connection between autoimmune thyroid diseases and bone metabolism

Common immune cells, along with shared cytokines and signaling pathways, play significant roles in both AITD and bone metabolism. Cytokines such as IL-6 and IFN-γ secreted by T lymphocytes are involved in the occurrence and progression of AITD and also exert dual effects on bone resorption. B lymphocytes are involved in AITD pathogenesis through antibody production and also modulate bone metabolism by affecting osteoclast and osteoblast formation. Furthermore, AITD-related antibodies can directly influence bone metabolism. Research has shown that TSHR is expressed on osteoclasts and osteoblasts (30). In GD patients, TRAb might suppress osteoblasts and osteoclasts by mimicking TSH activity. In GD patients, TRAb may mimic the action of TSH, not only inhibiting osteoclast formation and survival by attenuating JNK/c-jun and NFκB signaling but also suppressing osteoblast differentiation by downregulating Wnt (LRP-5) and VEGF (Flk) signaling (31). Thus, TRAb may exert a dual effect on bone metabolism through the mechanisms described above. Meanwhile, TSAb can also enhance the production of reactive oxygen species (ROS) by stimulating oxidative stress responses. ROS can facilitate osteoclast formation by engaging in signaling pathways related to osteoclastogenesis (e.g., NF-κB and MAPK pathways), elevating the risk of osteoporosis (32). Therefore, TRAb may influence bone metabolism through oxidative stress responses. Further research indicates that in GD mouse models, TRAb suppresses osteoblast differentiation by activating the AKT/mTOR signaling pathway, consequently promoting increased bone resorption (33). At present, the direct mechanisms through which TPOAb and TgAb influence bone metabolism remain unclear. Ruggeri et al. found that TPOAb in HT patients was significantly positively correlated with advanced glycation end products (AGEs, markers of oxidative stress) (3), and oxidative stress negatively correlates with bone mass by increasing the RANKL/OPG ratio and enhancing bone resorption (34). This suggests that TPOAb may influence bone metabolism through oxidative stress responses.

## 5 Summary

Epidemiological studies have indicated that the prevalence of TPOAb and TgAb positivity is 11.5% and 12.0%, respectively, with significantly higher rates observed in women compared to men (35). Shared immune signaling pathways and cytokines are involved in the pathogenesis of AITD and bone metabolism dysregulation. Antibodies associated with AITD may exert an influence on bone metabolism. However, the specific mechanisms through which these immune factors affect BMD have not been fully elucidated, and their contribution to bone metabolism remains controversial.

Mira Al Shoufy proposed that in GD patients, TRAb levels remain strongly associated with reduced BMD even when thyroid function is normalized, challenging the assumption that restoration of euthyroidism alone is sufficient to reverse bone loss (36). In light of the fact that over 30% of Chinese adults aged 40–49 have low bone mass (37), the National Health Commission has advised the public to pay attention to BMD screening from the age of 40 onwards. It is therefore worth exploring whether bone mineral density (BMD) screening in patients with autoimmune thyroid diseases (AITDs) should be initiated at an earlier age. Even among patients whose BMD falls within the normal range, health education and lifestyle interventions, such as increased sun exposure and vitamin D supplementation, may be beneficial. Future studies should incorporate prospective cohort studies and mechanistic investigations to clarify whether AITD-related immune factors induce significant changes in BMD, whether they can serve as independent predictors of osteoporosis, and whether reductions in antibody levels can improve BMD. Such research will provide an important theoretical foundation for the clinical management of bone metabolism in patients with AITD.
